# Processing Ceramic Proton Conductor Membranes for Use in Steam Electrolysis

**DOI:** 10.3390/membranes10110339

**Published:** 2020-11-12

**Authors:** Kwati Leonard, Wendelin Deibert, Mariya E. Ivanova, Wilhelm A. Meulenberg, Tatsumi Ishihara, Hiroshige Matsumoto

**Affiliations:** 1 International Institute for Carbon Neutral Energy Research (WPI-I2CNER), Kyushu University 744 Motooka, Nishiku, Fukuoka 819-0395, Japan; ishihara@cstf.kyushu-u.ac.jp (T.I.); matsumoto@i2cner.kyushu-u.ac.jp (H.M.); 2 Institute of Energy and Climate Research IEK-1, Forschungszentrum Jülich GmbH, 52425 Jülich, Germany; w.deibert@fz-juelich.de (W.D.); m.ivanova@fz-juelich.de (M.E.I.); w.a.meulenberg@fz-juelich.de (W.A.M.)

**Keywords:** proton-conducting oxide, steam electrolysis, hydrogen production, tape casting

## Abstract

Steam electrolysis constitutes a prospective technology for industrial-scale hydrogen production. The use of ceramic proton-conducting electrolytes is a beneficial option for lowering the operating temperature. However, a significant challenge with this type of electrolyte has been upscaling robust planar type devices. The fabrication of such multi-layered devices, usually via a tape casting process, requires careful control of individual layers’ shrinkages to prevent warping and cracks during sintering. The present work highlights the successful processing of 50 × 50 mm^2^ planar electrode-supported barium cerium yttrium zirconate BaZr_0.44_Ce_0.36_Y_0.2_O_2.9_ (BZCY(54)_8/9_2) half cells via a sequential tape casting approach. The sintering parameters of the half-cells were analyzed and adjusted to obtain defect-free half-cells with diminished warping. Suitably dense and gas-tight electrolyte layers are obtained after co-sintering at 1350 °C for 5 h. We then assembled an electrolysis cell using Ba_0.5_La_0.5_CoO_3−δ_ as the steam electrode, screen printed on the electrolyte layer, and fired at 800 °C. A typical Ba_0.5_La_0.5_CoO_3−δ_|BaZr_0.44_Ce_0.36_Y_0.2_O_3−δ_(15 μm)|NiO-SrZr_0.5_Ce_0.4_Y_0.1_O_3−δ_ cell at 600 °C with 80% steam in the anode compartment reached reproducible terminal voltages of 1.4 V @ 500 mA·cm^−2^, achieving ~84% Faradaic efficiency. Besides electrochemical characterization, the morphology and microstructure of the layered half-cells were analyzed by a combination of high-angle annular dark-field scanning transmission electron microscopy (HAADF-STEM) and energy-dispersive X-ray spectroscopy. Our results also provide a feasible approach for realizing the low-cost fabrication of large-sized protonic ceramic conducting electrolysis cells (PCECs).

## 1. Introduction

Steam electrolysis has been demonstrated to be an efficient and viable method to produce high purity hydrogen using ceramics proton-conducting electrolytes (PCE) at the intermediate temperature range (400~600 °C) [1,2,3,4,5]. This class of electrolytes is particularly favored for their relatively high ionic conductivity with low activation energy (<0.5 eV) for proton conduction [6,7]. Moreover, as protons are the conducting ionic species, hydrogen is produced separately from the supplied steam, which is a significant advantage compared to devices based on oxide-ion conducting electrolytes in which the produced hydrogen is obtained mixed with steam, requiring an additional separation process. There has been an increased interest in this technology in recent years, aimed at exploiting the benefits mentioned above [8,9]. Significant effort has been mostly focused on developing suitable electrolyte [6,7,10,11] and air electrode materials [12,13,14,15]. Whereas concerning device fabrication and assembly, progress has been made mainly with realizing small scale single cells having relatively small overall sizes and electrode surface area (0.2~0.4 cm^2^) [3,5,12,14,15,16,17,18]. For example, Duan et al. [2] recently reported a 90–98% Faradaic efficiency on a reversible protonic ceramic electrochemical cell operating endothermically with ~97% overall electric-to-hydrogen energy conversion efficiency at a current density of ~1000 mA·cm^−2^. Kim et al. [19] also obtain an attractive electrolysis voltage of 1.3 V at a current density of 750 mA·cm^−2^ and purporting the highest electrochemical performance for hydrogen production at 600 °C. Another remarkable achievement was reported by Wu et al. [20]. In their work, current densities of 210 and 880 mA·cm^−2^ at 500 and 600 °C, respectively, were obtained at an applied electrolysis voltage of 1.34 V. In our previous study, steam electrolysis voltage as low as 1.45 V was attainable at current densities of 0.2 and 0.5 A cm^−2^ at 550 and 600 °C, with Faradaic efficiency >82% using BaZr_0.44_Ce_0.36_Y_0.2_O_3−δ_ as the electrolyte [3]. The improved cell performance was explained by the electrolyte’s high densification promoted by a better sinter-ability of NiO-SrZr_0.5_Ce_0.4_Y_0.1_O_3−δ_ cathode substrate.

Although these small-sized protonic cells can successfully be deployed to reach very high hydrogen production performance, their fabrication techniques are not yet entirely rational at an industrial scale. Many factors can challenge the successful fabrication and upscale of affordable large-sized protonic devices meeting the commercial target. These large-sized devices are generally categorized as tubular or planar, with the planar type preferable for operation due to their advantages [21,22,23]. Nonetheless, the fabrication of such multi-layered planar devices, usually via a tape casting process, requires careful control of individual layers’ shrinkages to prevent bending and cracking during sintering [22,24,25]. In many cases, an additional flattening step is also necessary to minimize the bending caused by the layers’ different sintering behavior.

In recent years, various efforts to develop cost-effective processing techniques for large-sized protonic cells have somewhat advanced. Dailly and Marrony [26,27] reported on planar-type proton-conducting electrolyte cells with an active working area of ~12–20 cm^2^. In their study, an impressive cell performance on a 4.5 × 4.5 cm^2^ cell is reported under both fuel (>0.25 W /cm^2^, 0.7 V) and electrolysis mode (0.5 A/cm^2^, 1.3 V). They also observe stable long term operation and limited degradation rate (−2%/kh) [28]. Nonetheless, even with such a good performance, the investigated cells’ microstructure still requires significant optimization. Typically, for the successful fabrication of such devices with a relatively thin electrolyte layer, the fuel side electrode’s microstructural integrity is critical because its surface crucially affects the thin electrolyte layer’s quality. Bae et al. [29] and Himeko et al. [30] showed that over-grown NiO particles at the electrode interface tend to prevent a favorable microstructure at the electrolyte/electrode interface that contributes to the electrode reaction. A convenient, cost-effective processing method is thus needed to produce a desirable electrolyte/electrode interface microstructure and defect-free (without cracks, pinholes) thin electrolyte layer for better cell performance.

We focused on a well-established, low cost, sequential tape casting processing route to address some of these challenges. This advanced casting technique involves the inversion of the traditional tape casting approach’s necessary manufacturing steps and produces homogeneous, large-area, flat ceramic films. This method has been used for the fabrication of solid oxide-oxygen ions conducting fuel and electrolysis cell (O-SOFC/O-SOEC) [21,23] as well as hydrogen [31,32] and oxygen separation membranes [33,34,35]. Another great advantage of this technique is its potential to minimize manufacturing cost since multi-layer components are laminated and co-sintered in a single step [23,35]. Here, layered planar electrode-supported barium cerium yttrium zirconate BaZr_0.44_Ce_0.36_Y_0.2_O_3−δ_ half-cells were processed via a sequential tape casting route on a high shrinkage NiO-SZr_0.5_Ce_0.4_Y_0.1_O_3−δ_ support. The sintering parameters of the half-cells were analyzed and adjusted to obtain defect-free half-cells with diminished warping. Steam electrolysis cells were then assembled with Ba_0.5_La_0.5_CoO_3−δ_ as the steam electrode. The cells reached reproducible terminal voltages of 1.4 V at 500 mA·cm^−2^ with ~84 % hydrogen production efficiency.

## 2. Materials and Methods

Powders of BaZr_0.44_Ce_0.36_Y_0.2_O_3−δ_ (BZCY(54)_8/9_2), SrZr_0.5_Ce_0.4_Y_0.1_O_3−δ_ (SZCY541), and Ba_0.5_La_0.5_CoO_3−δ_ (BLC) were synthesized by a modified wet solution route in house, using pre-determined concentrations of analytic grade metal nitrates [7,36]. Stoichiometric amounts of the following precursor’s Ba(NO_3_)_2_ (Wako, Osaka, Japan, 99.9%), ZrO(NO_3_)_2_ (Aldrich, Tokyo, Japan, 99%), Sr(NO_3_)_2_ (Soekawa Chemicals, Tokyo, Japan 99.9 %), Ce(NO_3_)_3_·6H_2_O (Kanto Chemical Co., Tokyo, Japan, INC 99.99%), Co(NO_3_)_2_·6H_2_O (Kanto Chemical Co., Tokyo, Japan, INC 99.95%), La(NO_3_)_3_·6H_2_O (Wako, city and country, 99.9%) and Y(NO_3_)_3_·6H_2_O (Mitsuwa’s Pure Chemicals, Osaka, Japan, 99.9 %) were dissolved in deionized water. EDTA (Dojindo, Osaka, Japan, 99%) and citric acid (Wako, Osaka, Japan, 99%) were added as complexing agents in a molar ratio ereof 1:1.5:1.5.Ammonium hydroxide solution (Chameleon Regent, Osaka, Japan, 28.0% NH_3_ in H_2_O) was subsequently added to promote dissolution and adjust the mixture’s pH to ~9. A dark viscous gel is formed after stirring at 160 °C for about six hours, which was then pre-fired at 240 °C in a vacuum oven under adequate ventilation to form a solid black precursor. The powders were then calcined at 900 °C for 10 h in air and subsequently roll milled using zirconia balls with ethanol to produce uniform, submicrometer particles. The powders’ calcination was made again at 1100 and 1300 °C for BLC and the electrolyte, respectively. The synthesized powders’ phase purity was confirmed by X-ray diffraction (Rigaku Ultima IV) with Cu-Kα radiations (40 kV, 40 mA). Diffraction patterns were obtained in the 2θ range between 10° and 80° with a step size of 0.02°. The data were subsequently refined by the Rietveld method. Large quantities of BZCY(54)_8/9_2 and SZCY541powders were also purchased from KUSAKA RARE METAL PRODUCTS Japan. The particle size distribution was investigated by laser diffraction using a Malvern Mastersizer 3000 granulometry (Malvern, UK) equipped with a Hydro E.V. humid dispersion unit. Their specific surface area was determined using Brunauer–Emmett–Teller analysis with nitrogen as measurement gas. Powders morphology was examined in a scanning electron microscope (SEM FEG Tescan MIRA3, Brno, Czech Republic) and (STEM-EDS; JEOL JEM-ARM200F, Hitachi HD-2300A, Tokyo, Japan). The acronym BZCY(54)_8/9_2, stands for: Ba = 1, Zr = 0.5 × 8/9 = 0.44, Ce = 0.4 × 8/9 = 0.36, and Y = 0.2.

### 2.1. Fabrication of Electrode-Supported Half-Cells by Sequential Tape-Casting

The respective layers had different suitable slurry formulations for the green tapes. The basic BZCY(54)_8/9_2 electrolyte slurry composition was based on a previously optimized slurry composition for La_5.4_WO_12-δ_ (LaWO) membrane fabrication [31]. A two-step procedure was used for slurry preparation. Firstly, powders, solvent, dispersant, and a pore-forming agent (for the electrode support only) were mixed to form a suspension. In the second step, a binder and plasticizers were added in a suitable ratio to make the tape flexible and resistant. The solid loading of the electrolyte slurry was 25 vol%. For the support layer slurry, commercial NiO powder (Vogler, raw material) and BZCY(54)_8/9_2 (KUSAKA RARE METAL PRODUCTS Co., LTD, Kanagawa, Japan), or in-house synthesized with a ratio of NiO:BZCY(54)_8/9_2 = 60:40 in wt%, were dispersed in an ethanol and methyl ethyl ketone (M.E.K.) mixture together with Nuosperse FX9086 (Elementis Specialties, Inc., London, UK) as the dispersing agent. Polyvinyl butyral (Butvar PVB-98, Solutia Inc., St. Louis, MO, USA), Solusolv 2075 (Solutia Inc., St. Louis, MO, USA), and polyethyleneglycol PEG 400 (Merck Schuchardt, Hohenbrunn, Germany) were subsequently added as plasticizers and binder, respectively, to provide mechanical stability and flexibility to the green tape. Rice starch in a weight ratio of 5% or 15% to the solid load of the support slurry was added to produce a percolating porous structure after burning out. The mixture was homogenized adequately in a Thinky ARV310CE planetary mixer and left to rest for about 48 h to de-air and completely dissolve the binder before casting experiments. Similarly, the functional layer’s slurry consists of NiO and SZCY541 (KUSAKA RARE METAL PRODUCTS Japan) in a 60:40 weight ratio, without a pore-former, to increase the triple-phase boundaries and therefore the cathode activity.

Electrode-supported electrolyte half-cells were thus fabricated by sequential tape-casting using a KAROcast 300-7 micro-tape casting device from K.M.S. Automation GmbH Germany. The protocol consisted of; first, casting a thin BZCY(54)_8/9_2 electrolyte layer onto a silicone-coated polymeric (Polyethylene terephthalate) foil, moving with controlled speed into a vented instrument chamber. After proper drying at room temperature, a functional NiO-SZCY541 layer was cast directly on the BZCY(54)_8/9_2 film followed by the support NiO-SZCY541 or NiO-BZCY(54)_8/9_2 slurry with a 6-h interval for drying. This approach enables the formation of defect-free electrolyte layers because of the polymer foil’s high surface quality. The as-fabricated green tapes were subsequently cut into appropriate dimensions (e.g., round and square shapes) and sintered at 1350, 1400, and 1500 °C for further characterization. Co-firing consisted of de-binding to burn out the organic components and subsequent sintering to achieve the desired microstructure. The de-binding and sintering heating ramps were adjusted to obtain defect and crack free half-cells. The minimum electrolyte, functional, and support layer thickness was ~12, 10, and 400 μm, respectively (in the final-fired state).

Thermo-gravimetric analysis (TGA)/differential thermal analysis (DTA) was performed on tape caste BZCY(54)_8/9_2 single electrolyte and single support layer to understand the decomposition behavior of the organic additives, using a Netzsch S.T.A. 409C system. DTA involves detecting thermal effects accompanied by physical or chemical changes by recording the temperature difference (∆T) between the test sample and the reference sample. The measurements were performed in a constant flow of two gases: nitrogen and oxygen at ambient pressure with a heating rate of 5 °C /min up to a maximum temperature of 1000 °C. The bending action of the trilayer half-cell was investigated by first, comparing the shrinkage behavior of NiO-SZCY541 and NiO-BZCY(54)_8/9_2 with the BZCY(54)_8/9_2 electrolyte using a TOMMI plus optical dilatometer (Fraunhofer I.S.C., Würzburg, Germany). Samples for shrinkage measurements were prepared by cutting off strips with a width of 15 mm from the respective green tapes and shaping them into cylinders. Round shaped green tape pieces with a 28 mm diameter were used for bending behavior investigations, using heating ramps of 1~4 °C/min and temperatures up to 1500 °C with dwell times of up to 3 h. Images of the sample silhouette were recorded every 60 s with a charge-coupled device (C.C.D.) camera during heat treatment and analyzed subsequently. Finally, the gas-tightness of the electrolyte layer (50 × 50 mm^2^) was evaluated by helium leak rate measurements [23,37]. The helium flow through the half-cell was determined with a mass spectrometer at a pressure difference of 1000 hPa. The values were normalized to a measured area of 16 cm^2^ and a pressure difference of 100 hPa.

### 2.2. Complete Cell Fabrication and Steam Electrolysis Measurements

BLC electrode slurry was screen printed onto the BZCY(54)_8/9_2 electrolyte to form the complete cell with an active area of about 1 and 0.5 cm^2^ after firing at 800 °C/1 h. Silver-Palladium pastes and platinum wires were used as current collectors and leads, respectively. Steam electrolysis cells were mounted and sealed in a Probostat (NorECS, Oslo, Norway) using pyrex glass. Humidified 80% H_2_O mixed with 1% O_2_ / 99%Ar/ (30 mL·min^−1^) as the carrier gas was introduced to the anode and 1%H_2_ /Ar 99% (30 mL·min^−1^) to the cathode, humidified by saturated water vapor at 17 °C (*P*_H2O_ = 1.9 × 10^3^ Pa). A hygrometer chilled mirror (UHQ-4P, Buck Research Instruments LLC, Tokyo, Japan) was used to monitor the anode inlet’s water vapor pressure. The gas lines were kept heated at 120 °C to prevent the condensation of water vapor. The hydrogen production rate was determined by measuring the increase in hydrogen concentration in the cathode gas outlet by gas chromatography (Varian CP-4900 micro-Gas Chromatograph equipped with a micro-machined Thermal Conductivity Detector (T.C.D.), Agilent Technologies Inc., Tokyo, Japan). Faradaic efficiencies were evaluated based on the ratio between the experimental hydrogen generation rate and theoretical one at fixed current densities.

### 2.3. Microstructural Observation and Characterization

Scanning transmission electron microscopy–energy dispersive X-ray spectroscopy (STEM-EDS; JEOL JEM-ARM200F, Hitachi HD-2300A, Tokyo, Japan) was applied to analyze the top surface of the electrolyte, the half-cell as well, as the elemental distribution. Transmission electron microscopy (TEM.; JEOL JEM-ARM200F, Tokyo, Japan) was used to analyze the crystal structure. A focused ion beam coupled with a scanning electron microscope (FIB-SEM, Helios Nanolab 600i, Tokyo, Japan) was used to prepared TEM samples. Samples were first embedded in liquid epofix resin and a hardener, in a 9:1 ratio, and 0.4 W% methyl ethyl ketone added to improve the viscosity. Samples were left to harden overnight and subsequently polished to a mirror finish (0.25 μm) on one face using SiC grinding paper and water-based diamond suspensions sequentially. FIB was then used to obtain a micrometer-sized (~5 × 5 µm^2^) sample, which was machined to a thickness (<80 nm) suitable for observation. The tape cast half-cell structures were characterized in the x–z cross-section (x, tape-casting direction; z, thickness direction).

## 3. Results and Discussions

### 3.1. Phase and Structural Characterization of Powders

**Figure 1:**
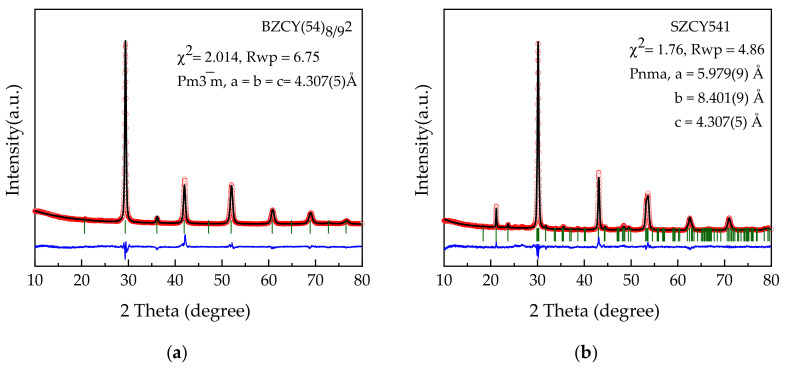
Rietveld refined XRD pattern of (**a**) BZCY(54)_8/9_2 powder and (**b**) SZCY541 powder after calcination at 1300 °C: observed X-ray diffraction intensity (black points) and calculated curve (red line). The bottom curve is the difference of the patterns, Yobs −Ycal, and the small greenish bars indicate the angular positions of the allowed Bragg reflections.

X-ray diffraction patterns obtained for BZCY(54)_8/9_2 and SZCY541 powders (in house synthesized) after calcination at 1300 °C are shown in Figure 1a,b together with derived Rietveld refinement parameters. The patterns clearly show dominant perovskite phases in agreement with previously reported results [3,7]. XRD results of the as-purchased powders calcined at 1200 °C also show a characteristic pattern similar to the latter and are presented in the supplementary Figure S1. Illustrated in Supplementary Figure S2 is the SEM morphology of both the purchased and in-house synthesized BZCY(54)_8/9_2 powders dispersed in the slurries used for tape-casting. The in house powder revealed sub-micrometric particles, with uniform morphology forming agglomerates of micrometers size. Whereas the purchase powder morphology seems slightly irregular with minor agglomerated particles. Laser diffraction and BET analysis estimate a mean particle size d_50_ of 0.65 µm and a specific surface area (A_spec_) of 1.9 m^2^/g for the in house synthesized BZCY(54)_8/9_2 powders, while for the purchased, these values were d_50_ of 0.72 µm and A_spec_ of 5.1 m^2^/g. These values are entirely satisfactory for making optimal tape casting slurries [31,37,38]. Table 1 depicts a summary of the powder characteristics.

### 3.2. Sequential Tape Casting and Characterization

Currently, tape casting is a proven technology for large-scale ceramic components with precisely controlled surface flatness. This versatile approach requires optimally formulated ceramic slurries [21,22,38]. Slurries with high solids loading and low viscosity are crucial for obtaining green tape with high density and homogeneous microstructure. After optimizing both the functional NiO-SZCY541 and substrate support NiO-SZCY541/NiO-BZCY(54)_8/9_2 layer slurries, half-cells were fabricated by sequential casting. First, a dense thin BZCY(54)_8/9_2 layer was cast on a polymer foil, using a 65 µm doctor blade gap. An approximately 6-h interval of drying in air at room temperature results in a defect-free layer with a homogenous thickness of ~16.5 µm in the green state. Subsequently, the functional and substrate support layers cast successively with similar drying intervals, respectively. We noted that pinholes and other defects are prone to appear on the electrolyte layer in case the underlying sheet is not adequately dried and a subsequent layer cast ontop. In such a case, the solvent from the cast layer either dissolves or seeps into the initial thin layer, thus resulting in defects in the tape, and in the end, fired half-cell.

**Figure 2:**
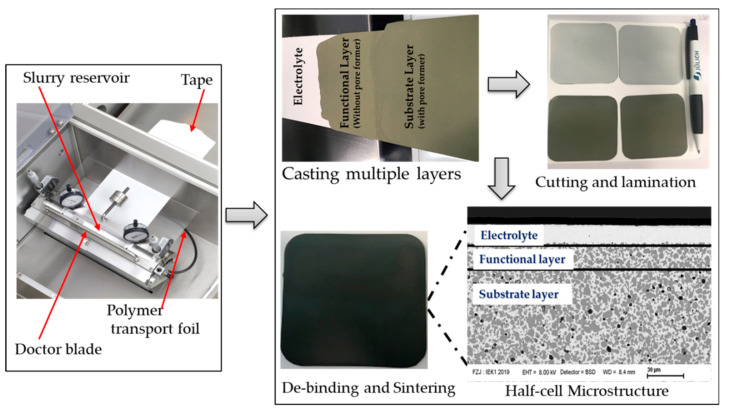
The sequential multi-layer processing technique of half-cell and the obtain microstructure after sintering at 1350 °C /5.

By controlling the doctor blade’s height, the electrolyte’s thickness was varied from 20 to ~12 μm. Flat, symmetrical 25 × 50 mm^2,^ and 50 × 50 mm^2^ planar half-cells, in the end, fired state and 22 mm in diameter, were routinely obtained via this procedure. Figure 2 depicts a short description of the processing technique. Adjusted casting speed, blade gabs, and some casting parameters are summarised in Table 2. The green tape produced was cut into different sample geometries; round shape ∅ 28 mm, by punching and square shape (65 × 65 mm^2^ and 130 × 130 mm^2^) using a scalpel blade for subsequent characterization. We noted that some of the samples cut using the scalpel blade resulted in non-uniform edges, which possibly led to additional sintering warpages, especially at the corners, as observed in some of the end fired samples.

**Figure 3:**
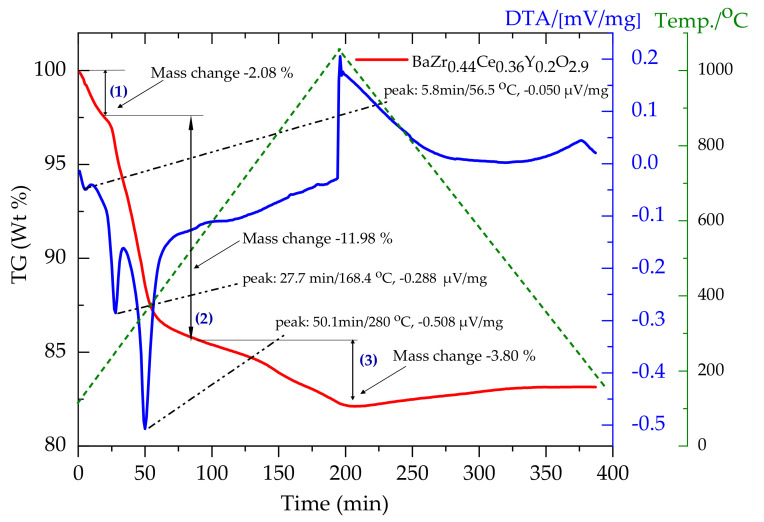
Thermogravimetric analysis (red curve)/differential thermal analysis (DTA blue curve) curve of BZCY(54)_8/9_2 electrolyte as measured in the coupled TG/DTA–MS system.

The co-sintering of multi-layer systems generally requires an appropriate temperature program to avoid the creation and propagation of defects while the removal of the organic additive takes place. A proper understanding of the organic additive’s thermal behavior is essential to minimize undesirable defects such as cracks, warpage, or delamination occurring during sintering. Hence, a suitable heating schedule and sufficient holding time at every intermediate stage are necessary to prevent the latter. For this purpose, simultaneous TG/DTA coupled to quadrupole mass spectrometer was performed on the green tapes to understand the organic additives’ decomposition behavior. Measurements were performed on the BZCY(54)_8/9_2 electrolyte, NiO-SZCY541, and NiO-BZCY(54)_8/9_2 single layers. Figure 3 presents TG/DTA curves for the BZCY(54)_8/9_2 single electrolyte layer. The curve reveals three successive distinct regions of mass changes corresponding to a total mass loss of 17.9% recorded in the temperature range ~56.5 °C to 480 °C, beyond which no further mass loss was observed up to 1000 °C. The first region connects with a DTA endothermic trench down to 56.5 °C, which may be associated with the release of residual solvents (and/or a minute amount of adsorbed water on the surface of the tape). The second is connected with two endothermic peaks with maxima at 168 and 280 °C associated with an intensive release of organic additives [39,40,41,42]. These temperature ranges happen to also correspond with reported regimes (~270 °C–340 °C and 340 °C–400 °C) for PEG-400, and PVB-98 burnout in green tapes, respectively [37,38,39,40].

**Figure 4:**
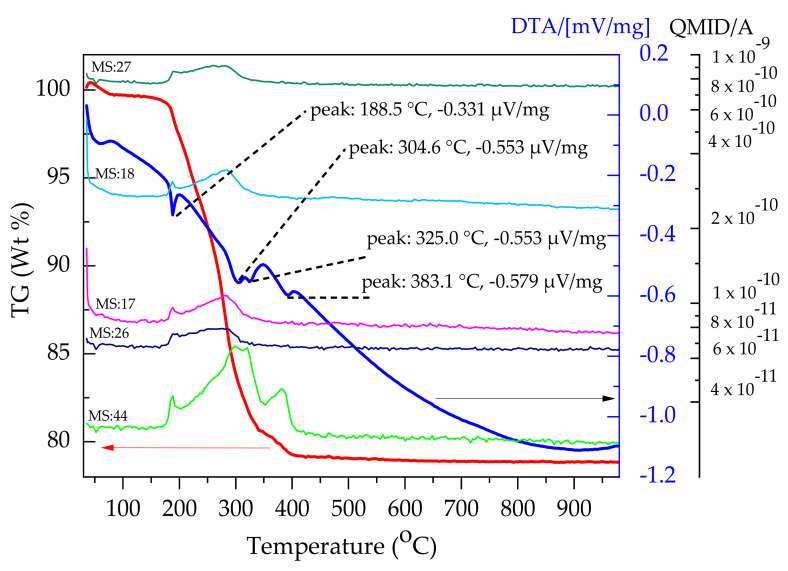
Thermogravimetric mass spectrometric coupled analyses of NiO-BZCY(54)_8/9_2 layer TG, DTA curves with the total ion current plot of the evolved residues.

For a better comprehension of the evolved residual components during the thermal decomposition, TG/DTA–MS analyses carried out on NiO-BZCY(54)_8/9_2 single electrode layer is presented in Figure 4. As observed, the weight loss increases slowly up to ~174 °C, after which a sharp increase of ~20 % in the temperature range 175–404 °C appeared. The DTA curve shows four major endothermic peaks trenching down to 189, 305, 325, and 383 °C. The first three peaks connect with MS spectra of *m*/*z* = 17, 18, 26, 27, and 44 ion fragments. The *m*/*z* = 17 (OH^+^), 18, are the characteristic MS values for H_2_O. Similarly, the *m*/*z* 44 (CO_2_^+^) curve is indicative of the possible release of CO_2_. TG/DTA–MS analyses carried out on NiO-SZCY541 single layers showed similar burn out characteristics and presented similar MS signal patterns with common *m*/*z* ions fragments (data not shown).

Based on the above thermogravimetric analysis in Figure 3 and Figure 4, a sintering profile for the half-cells was formulated with a 1 °C /min heating rate. Subsequently, a holding stage at 300 °C for 30 min to sufficiently burn out the additives and another at 600 °C to complete the decomposition of the residual polymer.

### 3.3. Sinterability and Bending Behavior of the Half-Cell

Lowering the sintering temperature of the BaZr_1-x-y_Ce_x_Y_y_O_3−δ_ (BZCY) based half-cell below 1300 °C is our utmost desire to limit Ba loss via evaporation during sintering. Ba loss is expected to be detrimental to electrolyte performance, as the conductivity of BZCY is known to be highly sensitive to Ba content [7,43]. A suitable electrode support, in principle, plays a crucial role in determining the co-sintering temperature at which the electrolyte layer is fully densified. In our previous study, we found that NiO-SrZr_0.5_Ce_0.4_Y_0.1_O_3−δ_ electrode substrate positively influenced the densification of BZCY(54)_8/9_2 layer on a 20 mm diameter half-cell configuration. Nonetheless, the co-firing of planar multi-layered half-cells are often more challenging due to the constituent layers’ different sintering behavior. The properties of the co-sintered individual layers are inevitably affected by stresses due to shrinkage mismatch at the interfaces as a result of the different thermal expansion of each segment and different sintering kinetics. Such stress could create processing defects like micro-cracks, warpage, or delamination at the interface between the layers. The sintering shrinkage of the NiO-SZCY541, NiO-BZCY(54)_8/9_2 substrates, and the half-cells were obtained from the height reduction of the cylinders [31,44]. Both composites display different shrinkage behaviors, exhibiting a maximum shrinkage rate at ~1350 °C for NiO-SZCY541 and 1450 °C for NiO-BZCY(54)_8/9_2, consistent with our previous work [3]. The NiO-SZCY541 and NiO-BZCY(54)_8/9_2 half-cell shrunk about 27 and ~33 %, respectively after sintering at 1500 °C, whereas the BZCY(54)_8/9_2 electrolyte only layer shrunk ~9.8 after sintering at the same temperature.

**Figure 5:**
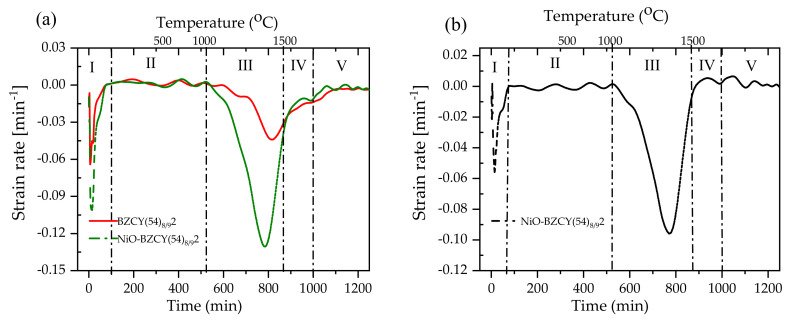
(**a**) Strain rates of the tape-casted BZCY(54)_8/9_2 and NiO-BZCY(54)_8/9_2 support substrates (**b**) Mismatch of strain rates (the difference between individual strain rates BZCY(54)_8/9_2 and NiO-BZCY(54)_8/9_2) between BZCY(54)_8/9_2 and NiO-BZCY(54)_8/9_2 support substrates determined as the net difference between the curves in Figure 5.

**Figure 6:**
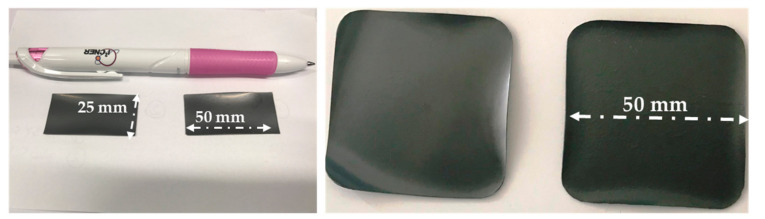
Images of some of the initial 25 × 50 mm^2^ and 50 × 50 mm^2^ planar BZCY(54)_8/9_2 based half-cells, in the end, fired state showing warpage, especially pronounced at the edges.

Figure 5 shows a plot of the shrinkage rates $d(∆l/l_{0})/dt$ as a function of time and temperature obtained from the change in cylinder height for BZCY(54)_8/9_2 and NiO-BZCY(54)_8/9_2, respectively. Five respective regions are distinguished (denoted as I, II, III, IV, and V), representing the sintering process during heating and cooling. Region (III) represents the primary area where sintering is dominant, although sintering continues throughout the dwell time (IV) at 1500 °C. While the initial sharp strain observed in the region (I) could be attributed to possible shrinkage due to the rapid release of residual solvents and a small amount of surface absorbed water, as discussed above. There was practically no difference in the sintering behavior between the two specimens in region II. The red curve designates the BZCY(54)_8/9_2 electrolyte, while the green represents the NiO-BZCY(54)_8/9_2 electrode substrate. The NiO-BZCY(54)_8/9_2 substrate exhibited a higher sintering rate than that of the BZCY(54)_8/9_2 electrolyte during thermal treatment. Figure 5b shows the mismatch of the sintering rates represented by the difference between the individual sintering rates of BZCY(54)_8/9_2 electrolyte and NiO-BZCY(54)_8/9_2. The stresses generated by the different sintering rates are the primary cause for the deformation of the half-cells, precisely the warpage or bending, observed upon co-firing in this work. The difference in the densification behavior is primarily due to the NiO phase in the composite substrate. NiO particles possibly form a constrained matrix surrounding BZCY(54)_8/9_2 particles, thus imposing compressive stress on them, allowing them to shrink faster [28,42]. Cai et al. [45,46,47] made calculations on the tensions that arise during densification of a bi-layer and concluded that bi-layers would, in principle, curve towards the layer that shrinks faster, with the degree of curvature, *k*, due to mismatch of the shrinkage rates expressed using the following equation:

(1) $$k=\frac{t_{1}+t_{2}}{r}=\frac{6{\left(m+1\right)}^{2}mn}{m^{4}n^{2}+2mn\left(2m^{2}+3m+2\right)+1}\cdot ∆έ$$

where *t*_1_ and *t*_2_ are the thickness of layers 1 and 2, *r* the radius of curvature, ∆έ is the strain rate mismatch between the layers, *m* the ratio of layer thickness, *n* the viscosity ratio between the layers. As expected, the BZCY(54)_8/9_2/NiO-BZCY(54)_8/9_2 half-cells could not be obtained without an extreme warping, especially pronounced at the edges for most of the end fired specimens. Figure 6 shows images of some 50 × 50 mm^2^ planar BZCY(54)_8/9_2 based half-cells with a 30% solid loading of the NiO-BZCY(54)_8/9_2 electrode substrate. As observed, the half-cells all appear curved toward the electrolyte top layer with warped edges. A much stronger curvature was systematically observed, with lower solid loading of the substrate. It is essential to note here that all other processing parameters were initially optimized and fixed at the same conditions except for the substrate support’s solid loading.

**Figure 7:**
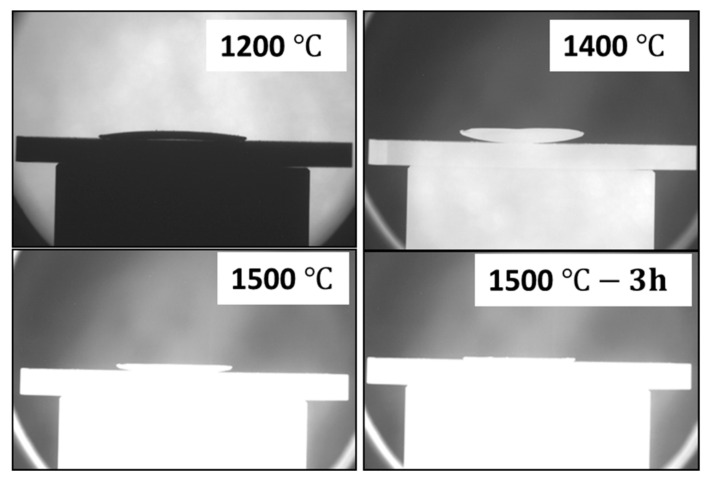
Selected sequence showing the curvature developed upon co-firing the BZCY(54)_8/9_2 half-cell taken with the optical dilatometer. (Electrolyte layer is facing upward with the NiO- BZCY(54)_8/9_2 underneath).

Therefore, the change of the curvature and warpage is mainly attributed to the variation in solid loading. The observed warpage and bending are due to strain rate mismatch between the layers and likely due to non-uniform green tape edges, obtained using the scalpel blade while cutting. To establish a suitable sintering profile that enables the preparation of flat half-cells with the desired microstructure. The thermal behavior of the half cells was further investigated in detail using optical dilatometry. Figure 7 shows a sequence of selected photographs of the shape evolution and bending behavior of the half-cell during sintering (with the BZCY(54)_8/9_2 layer facing upwards). For all the samples investigated, no apparent bending is observed within the initial stages of sintering until around 1000~1200 °C, where a slightly convex shape occurred with the half-cell curving towards the NiO-BZCY(54)_8/9_2 support side. This may result from the NiO-BZCY(54)8/92 support faster sintering compared to the BZCY(54)_8/9_2 electrolyte layer, consistent with the above discussion. This shape remains up to about 1250 °C, where the curvature rate inverts. The half-cells begin to curve towards the electrolyte side. The most substantial curvature toward the electrolyte layer occurred at a temperature of about 1400 °C. With further heat treatment to ~1500 °C, the observed bending begins to be compensated mainly by the NiO-BZCY(54)_8/9_2 electrode substrate. After a dwelling period of 3 h at 1500 °C, the half-cell is entirely flat and retains this configuration up to the end of the sintering process. This curvature evolution of the BZCY(54)_8/9_2 based half-cell seems consistent with in-situ observations made by Costa and Ravi et al. [22,48].

**Figure 8:**
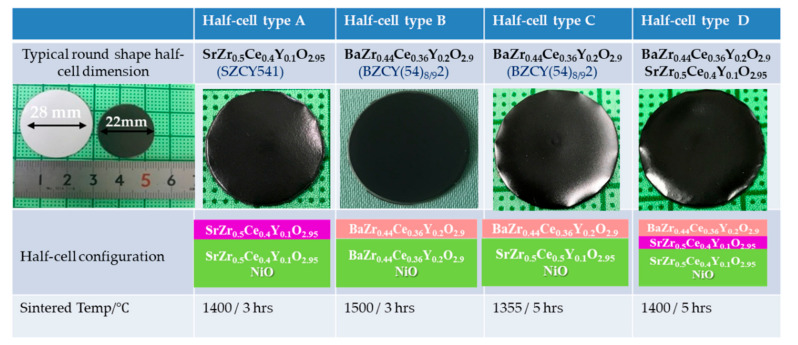
Photographic images of the as-fabricated round shape half-cell with different configurations (half-cell type A~D), typical dimension before and after sintering, and the sintering temperature.

However, we did not observe the BZCY(54)_8/9_2 electrolyte’s initial sintering as they did. The solid loading variation revealed an optimum NiO-BZCY(54)_8/9_2 solid loading of 31 vol%. Based on the above investigations, a slow heating rate and the introduction of a dense functional layer were very beneficial for limiting the developed curvature during the sintering process. However, for the planer type cells, the optimum solid substrate loading and sintering profile weren’t enough to obtain planar cathode-supported half-cells. Curvature formation still occurs though crack and warpage issues were eliminated. Therefore to control the half-cells’ planarity, the green cathode substrate thickness was increased from ~400 μm to 500 μm and resulted in flatting at the end fired state. By increasing, the cathode thickness, the planar half-cell strength is enhanced, and thus the overall half-cell curvature mitigated. Figure 8 shows photographic images of the as-fabricated round shape half-cell with different configuration (half-cell type A~D) and typical dimension before and after sintering. Each cell type is also described in the Figure and differ mostly at the cathode substrate. The half-cell remained flat after sintering without camber or warping for half-cell type B. Half-cell A, C, and D, even though somewhat flat, however, continue to show pronounced edge warpage, typically bending toward the substrate. The observation contrasts the edge warpage demonstrated by the 25 × 50 mm^2^ and 50 × 50 mm^2^ planar BZCY(54)_8/9_2 based half-cells shown in Figure 6. The edge warpage is slightly reduced for half-cell type C when sintered at 1350 °C. More detailed experimental investigation and numerical calculation models are still necessary to thoroughly understand the curvature evolution of BZCY based half-cells during sintering. Another noticeable observation was that the sintering mismatch is most pronounced for the configuration based on the NiO-SZCY541 electrode and BZCY(54)_8/9_2 electrolyte, inferring that the co-shrinking NiO-SZCY541 support influences the densification behavior of BZCY(54)_8/9_2 significantly. Suitably dense and gas-tight BZCY(54)_8/9_2 layers are obtained after co-sintering with NiO-SZCY541 at 1350 °C, whereas the densification of the tri-layered NiO-BZCY(54)_8/9_2 based half-cell typically occurred at 1450 °C. The co-sintered layers’ densities also exceeded the density of the BZCY(54)_8/9_2 free layers at 1450 °C.

The observed sintering evolution behavior was reproduced with the planar half-cells (65 × 65 mm × 0.530 mm), including the NiO-SZCY541 functional layer. Leak rate testing of the half-cells with helium showed an excellent gas tightness for both the sintered flat 22 mm diameter and 50 × 50 mm^2^ planar electrolyte layers. With an average leak rate of 5.17 × 10^–5^ and 4.41 × 10^–6^ hPa dm^3^ (s·cm^2^)^–1^ for BZCY(54)_8/9_2 sintered at 1355 and 1400 °C, respectively. Moreover, planar SZCY541, 50 × 50 mm^2^ half-cells generally showed better gas tightness, with an average leak rate of 6.83 × 10^–6^ hPa dm^3^ (s·cm^2^)^–1^ after sintering at 1400 °C. These leak rate values are comparable and well within the threshold for high-quality reported SOEC and SOFC gas-tight electrolytes [21,23].

### 3.4. Microstructure and Surface Morphology

**Figure 9:**
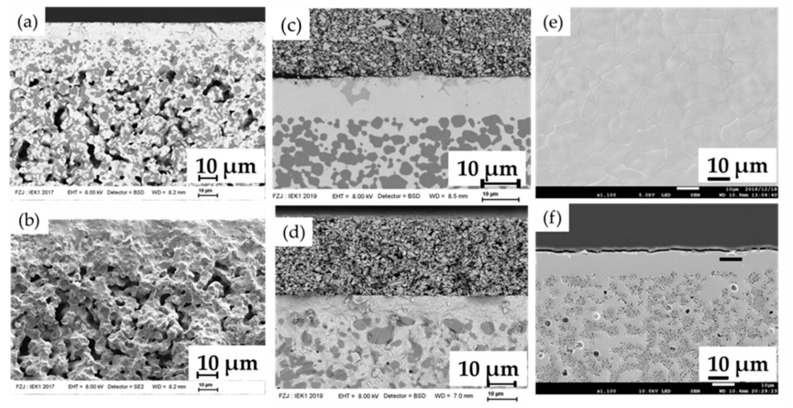
Scanning electron microscopy images of the as-fabricated BZCY(54)_8/9_2 electrolyte based cell. (**a**) BSD-SEM observation of a fracture cross-section of the BZCY(54)_8/9_2/NiO-SZCY541/ NiO-BZCY(54)_8/9_2 half-cell. (**b**) Fracture cross-section of the NiO-BZCY(54)_8/9_2/NiO-SZCY541/BZCY(54)_8/9_2 half-cell. (**c**,**d**) The microstructure of a single cell composed of BLC electrode anode (half-cell sintered at 1450 and 1350 °C respectively) (**e**) Typical surface view of the BZCY(54)_8/9_2 layer after sintering. (**f**) polished cross-section of a half-cell after NiO reduction.

Figure 9 shows the cross-section and surface morphology of some selected half and single cells. A well-organized, multi-layered structure without visible deformation, delamination, or cracks is observed (Figure 9a,b). The resulting NiO-SZCY541 electrode functional layer and BZCY(54)8/92 electrolyte layer are homogeneous with estimated thicknesses of about 10 and 15 µm, respectively, after sintering at 1355 °C. The NiO-SZCY541 layer adheres well to the BZCY(54)_8/9_2 layer and forms an excellent percolating network, which should, in principle, lead to more triple-phase boundaries accessible for charge species and, thus, is expected to enhance the cathodic reaction. Figure 9c presents a complete cell’s typical microstructure with the half-cell sintered at 1450 °C for 5 hrs. The NiO-BZCY(54)_8/9_2 substrate layer, the NiO-SZCY54 functional layer, the BZCY(54)_8/9_2 electrolyte, and the steam electrode layers (BLC) each adheres well to the adjacent layer after sintering. However, the BZCY(54_)8/9_2 electrolyte phase’s systematic contamination by secondary phase precipitates of yttrium-rich barium nickel oxides was observed (see Figure S3). The contamination is even more severe at 1500 °C, mostly distributed at the interfaces and on the outer surface. A possible reason for this behavior could be the A-site substitution of Ba atoms by Ni and Y species (BaY_2_NiO_5_ phase) during sintering [49,50]. Ba loss via evaporation is known to occur during sintering in air, and the loss amount turns to increases with increasing sintering temperature [41]. Lee et al. [51] have also reported on the formation BaY_2_NiO_5_ phase in BZCY based electrolyte and suggested that it assist the electrolyte densification. Figure 9d shows a cross-sectional view of a fractured type C single-cell. As observed, the different layers also adhered well to each other, and the electrolyte layer is dense and free of pinholes. Figure 9e also displays that the BZCY(54)_8/9_2 electrolyte layer is fully dense, and the grain size is in micron-scale and about 7~8 μm with no pores. To understand the chemical compositions of the as fabricated half-cells, EDS and STEM investigations were performed on half-cell sintered at 1500 °C.

**Figure 10:**
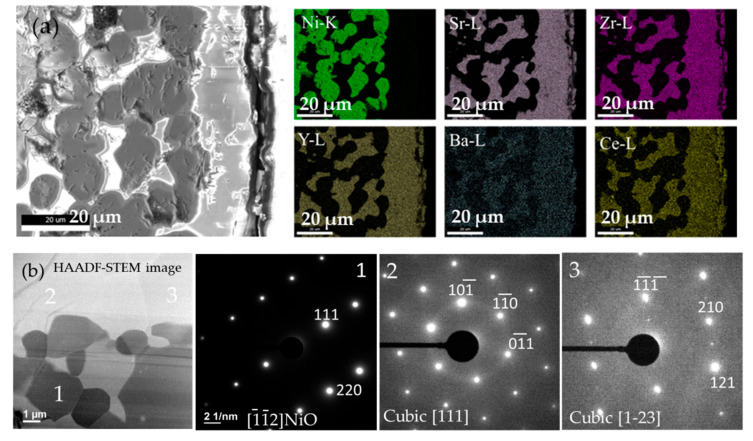
(**a**) SEM and STEM analysis of NiO-BZCY(54)_8/9_2/NiO-SZCY541/BZCY(54)_8/9_2 half-cell with corresponding EDS maps of Ni, Sr, Zr, Y, Ba, Ce. (**b**) High-angle annular dark-field (HAADF) micrograph of the BZCY(54)_8/9_2/NiO-BZCY(54)_8/9_2 support substrate interface and selected-area electron diffraction pattern. The Miller indices and crystallographic direction labeled are those of Ni and BZCY(54)_8/9_2.

Figure 10 shows the SEM image of a polished cross-section of the BZCY(54)_8/9_2/NiO-SZCY541/ NiO-BZCY(54)_8/9_2 half-cell with the corresponding EDS elemental maps of Ni, Sr, Zr, Y, Ba, Ce. However, the maps confirm no perceivable Ni diffusion from the substrate to the BZCY(54)_8/9_2 electrolyte, at least within the instrument resolution limits. The maps further reveal Sr is predominantly present and uniformly distributed throughout the BZCY(54)_8/9_2 electrolyte, overlapping with Ba, thus suggesting significant Sr segregation upon sintering at 1500 °C. The Ni area also reveals the presence of Ba spread entirely throughout the observed region. The spreading of elements is attributed primarily to barium and cerium’s evaporation from the BZCY(54)_8/9_2 phase during sintering at 1500 °C. Ba evaporation in BZCY(54)_8/9_2 is consistent with common observations in barium cerate and zirconate based materials that invariably experienced deficiency in the Ba site after high-temperature sintering [41,47,48].

A substantial amount of Ba loss is also observed, as indicated by the color contrast in the Ba map. The EDS maps for the sample sintered at 1355 °C (data not shown) indicate that the element distribution is somewhat more homogeneous and that no significant elemental enrichment exists in BZCY(54)_8/9_2/NiO-SZCY541/NiO-BZCY(54)_8/9_2 matrix entirely. More detailed microstructural observations were made via selected-area electron diffraction (SAED). Figure 10b show SAED patterns taken from the BZCY(54)_8/9_2 /NiO-BZCY(54)_8/9_2 support on the Ni and BZCY(54)_8/9_2 surface (spot 1, 2, and 3). A high-resolution TEM (HRTEM) image of area 1 (Figure 10b) resolved the [111] and [220] lattice fringes along the [112] zone axis of the Ni. Spot 2 and 3 viewed along the [111] and [123] direction showed lattice planes with a 0.419 nm spacing attributed to the cubic BZCY(54)_8/9_2 structure. This is consistent with the XRD spectrum acquired from the same sample.

### 3.5. Single-Cell Performance

**Figure 11:**
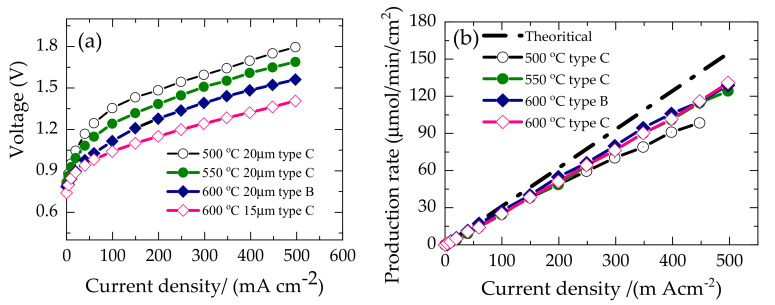
(**a**) Temperature dependence I–V characteristic of the as-fabricated steam electrolysis cells based on Type B and C half-cell configuration (**b**) Hydrogen evolution rate measured at 500, 550, and 600 °C.

To investigate the steam electrolysis performance, BLC was chosen as the steam electrode and applied onto the dense electrolyte surface by screen printing followed by sintering at 800 °C for 1 h. Figure 11 shows typical I–V characteristics of single-cells made from type B and C based half-cells, BLC|BZCY(54)_8/9_2|Ni-BZCY(54)_8/9_2 and BLC|BZCY(54)_8/9_2|Ni-SZCY54 under the supply of humidified 80% H_2_O diluted with 1% O_2_ / 99%Ar to the anode and 1% H_2_ /Ar 99% at the cathode as the sweep gas. The former cell configuration’s open-circuit voltage was 0.79 V at 600 °C, verifying that the electrolyte layer was dense enough to ensure sufficient gas tightness.

Voltages of 0.75, 0.82, and 0.80 V were obtained from the latter at 600, 550, and 500 °C, respectively. However, the measured values are slightly lower than the theoretically obtained values of 0.83, 0.86, and 0.88 V at 600, 550, and 500 °C, from the Nernst equilibrium equation (Equation (2)) shown below for a proton conductor under these gas atmospheres. This deviation is due to limited p-type electronic conductivity in the BZCY(54)_8/9_2 electrolyte layer [7,52,53,54], with the possible existence of minute pinholes on the electrolyte layer, facilitating little cathode/gas crossflow.

(2) $$E=\frac{RT}{2F}\ln\left(\frac{PH_{2,\;cathode}}{PH_{2,\;anode}}\right)=\frac{∆G^{o}}{2F}+\frac{RT}{2F}\ln\left(\frac{PO_{2,anode}^{0.5}\cdot PH_{2,\;cathode}}{PH_{2}O_{\;anode}}\right)$$

The Type B half-cell based configuration’s electrolysis current density reached 0.5 A cm^−2^ with an applied voltage of 1.56 at 600 °C. In contrast, the Type C design attains 0.5 A cm^−2^ with applied voltages of 1.79, 1.68, 1.40 V at 500, 550, and 600 °C, respectively. The overall cell overpotential decreased processively with an increase in temperature from 500 to 600 °C, as shown in Figure 11a. A distinct non-linear *V–I* slope behavior is exhibited at low current densities for all the cells up to about 100 mA /cm^2^, with high activation overpotential. Hence, indicating a more sluggish electrode reaction with decreasing temperature. The thermal-neutral voltage for steam electrolysis, defined as the voltage at which the joule heating from the cell is equivalent to the endothermic heat demand, is 1.28 V at 600 °C. Within this approximately applied voltage regime in Figure 11a, the initial I-V curvature begins to shift to a constant slope. This slope change phenomenon could also be related to the cell’s cooling via the endothermic energy demand [52,55]. It occurs ~100 mA/cm^2^ at 600 °C for Type B and C based cell configuration. An effective electrolyzer must convert a high fraction of the current into hydrogen; in other words, it should have high faradaic efficiency. The amount of hydrogen gas evolved at the cathode on passing a direct current through the cells was confirmed by gas chromatography. Figure 11b presents the hydrogen evolution rate measured at 500, 550, and 600 °C versus current density.

The black dash line represents the theoretical evolution rate estimated from Faraday’s law. The hydrogen evolution rate follows Faraday’s law and is proportional to the current density even though deviations are noted at higher current densities. The faradaic efficiency was 84 and 82% at 500 m Acm^−2^ for the Type B and C based cell configuration at 600 °C, deviating from the faradaic efficiency. The deviation becomes more pronounced at 500 and 550 °C. These substantial deviations are due to electronic current leakage through the BZCY(54)_8/9_2 electrolyte. It is well documented that the electronic transference number of ceramic proton conductors increases with increasing temperature and oxygen partial pressure [16,56] and can, in principle, lower the current efficiency. Another plausible reason for the low current efficiency at 500 °C could be the anode material BLC’s limited catalytic activity. The electrical energy efficiency of steam electrolysis, which is the amount of electrical energy input per unit of hydrogen produced, can be calculated using the following expression [3,57]:

(3) $$\mathrm{Electrical}\;\mathrm{energy}\;\mathrm{efficiency}(\eta_{EE})=\frac{Amount\;ofH_{2}\;production\times LHVofH_{2}}{Electrical\;energy\;imput}\times \left(FE\right)\times 100\;\%$$

(4) $$\eta_{EE}=\frac{∆H}{2FV}=\frac{1.28\;volts}{V}\times \left(FE\right)\times 100\;\%$$

where *EE* is the energy efficiency, *FE* is the faradaic efficiency, Δ*H* is the enthalpy change of hydrogen (246.69 kJ/mol at 600 °C), and *V* is the applied voltage, *F* is the Faraday constant. The electrical energy input per unit of hydrogen produced for the BZCY(54)_8/9_2 cell at 500 m Acm^−2^ with an applied voltage of 1.4 V is ~77 % (HHV standard). The amount of electricity required to produce 1 Nm^3^ of hydrogen is ~3.9 kWh ((1000 L Nm^−3^)/(22.4 L mol^−1^) × 2*F*/(3600 s h^−1^) × (1.41 V)/ 0.84))(Wh). These results clearly demonstrated that sequential tape casting is a feasible approach for realizing the low-cost fabrication of high performing P-SOECs.

## 4. Conclusions

In summary, flat tri-layer 50 × 50 mm^2^ planar barium cerium yttrium zirconate (BZCY(54)_8/9_2) half cells were processed via a cost-effective, scalable, sequential tape casting route. The shrinkage of the NiO-SZr_0.5_Ce_0.4_Y_0.1_O_2.95_ support during co-sintering uniformly promotes the densification of the BZCY(54)_8/9_2 electrolyte layer, thereby resulting in a remarkably reduced temperature of 1350 °C. Furthermore, a typical BZCY(54)_8/9_2 based single cell at 600 °C reached reproducible terminal voltages of 1.4 V @ 500 mA cm^−2^, achieving ~84 % faradaic efficiency regarding hydrogen production. Based on these results, the calculated amount of electricity to produce 1 Nm^3^ of H_2_ is ~3.9 kWh. Additional performance improvement is possible by further reducing the BZCY(54)_8/9_2 electrolyte thickness, exploring more efficient electrode materials, and optimizing the cell microstructure. Future work will focus on the cells’ characterization with an active area of 20 cm^2^ and strategies to mitigate electronic current leakages in protonic electrolysis cells.

## Figures and Tables

**Table 1:** Physical properties of the ceramic powders used for tape casting and processing.

Powder	Particle Diameter			Specific Surface Area A_spec_ (m^2^/g)	Calcination Temperature
	d_10_	d_50_	d_90_		
BZCY(54)_8/9_2 purchased	0.37	0.72	1.93	5.1	1200 °C
SZCY541 purchased	0.50	0.69	0.95	3.1	1200 °C
BZCY(54)_8/9_2 in house	0.41	0.65	1.12	1.9	1300 °C
SZCY541 in house	0.50	0.78	1.25	1.8	1300 °C

**Table 2:** Summary of the tape-casting parameters, Adjusted casting speed, and blade gaps used for fabrication of the proton-conducting half-cells.

Layer	Blade Gap [μm]	Casting Speed [mm/s]	Drying Time [h]	Green Thickness [μm]	End Fired Thickness [μm]	Investigation
**Electrolyte**						
Electrolyte 1	100	5	6	25	~20	TG/Shrinkage
Electrolyte 2	75	5	5	18.8	~15	Half-cell
Electrolyte 3	55	5	5	13.4	~10	Half-cell
**Substrate and functional layer (CFL)**						
CFL	40	10	5	10	~8	Half-cell
Substrate	1200	2.5	~10–12	502	~390–400	Half-cell

## Supplementary Materials

The following are available online at https://www.mdpi.com/2077-0375/10/11/339/s1, Figure S1: X-ray diffraction patterns of commercial SZCY541 and BZCY(54)_8/9_2 powders from KUSAKA RARE METAL PRODUCTS Co., LTD, Kanagawa, Japan. Figure S2: SEM morphology of BZCY(54)_8/9_2 powders (a) Purchased powder from KUSAKA RARE METAL PRODUCTS Co., LTD, Kanagawa, Japan, as calcined at 1200 °C (b) In house synthesized after calcination at 1300 °C and milling, FS3: XRD patterns of green NiO-BZCY(54)_8/9_2 electrode substrate and after sintering at 1350 and 1450 °C, Video S1: Shape evolution and bending behavior of the half-cell during sintering.
