# Supplementary material for: Processing Ceramic Proton Conductor Membranes for Use in Steam Electrolysis
Source: Membranes (Basel). 2020 Nov 12;10(11):339. doi: 10.3390/membranes10110339 (PMC7696768; doi:10.3390/membranes10110339)
Supplement: Supplementary file 1 [file membranes-10-00339-s001.zip › supplememtary final/Supplememtary Material.pdf]

Supplementary Materials

Processing Ceramic Proton Conductor Membranes for Use in Steam Electrolysis

Kwati Leonard [1,2,*], Wendelin Deibert [2], Mariya E. Ivanova [2], Wilhelm A. Meulenberg [2], Tatsumi Ishihara [1], and Hiroshige Matsumoto [1]

[1] International Institute for Carbon Neutral Energy Research (WPI-I²CNER), Kyushu University 744 Motooka, Nishiku, Fukuoka 819-0395, Japan; ishihara@cstf.kyushu-u.ac.jp (T.I.); matsumoto@i2cner.kyushu-u.ac.jp (H.M.)

[2] Institute of Energy and Climate Research IEK-1, Forschungszentrum Jülich GmbH, 52425 Jülich, Germany; w.deibert@fz-juelich.de (W.D.); m.ivanova@fz-juelich.de (M.E.I.); w.a.meulenberg@fz-juelich.de (W.A.M.)

* Correspondence: kwati@i2cner.kyushu-u.ac.jp; Tel.: +81-092-802-6717

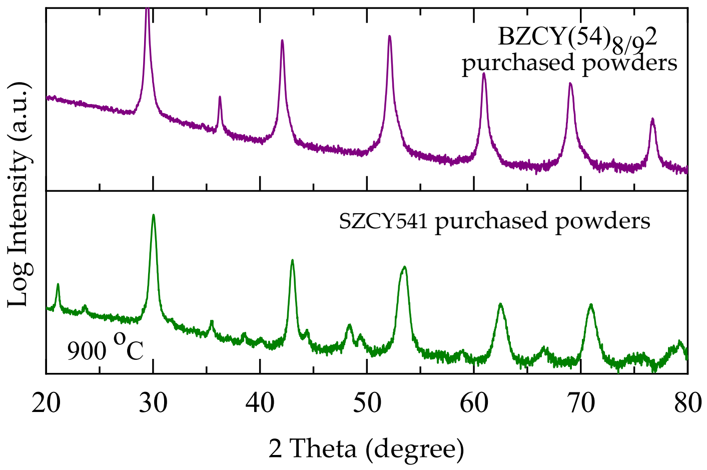

Figure S1. X-ray diffraction patterns of commercial SZCY541 and BZCY(54)$_{8/9}$2 powders from KUSAKA RARE METAL PRODUCTS Co., LTD, Japan

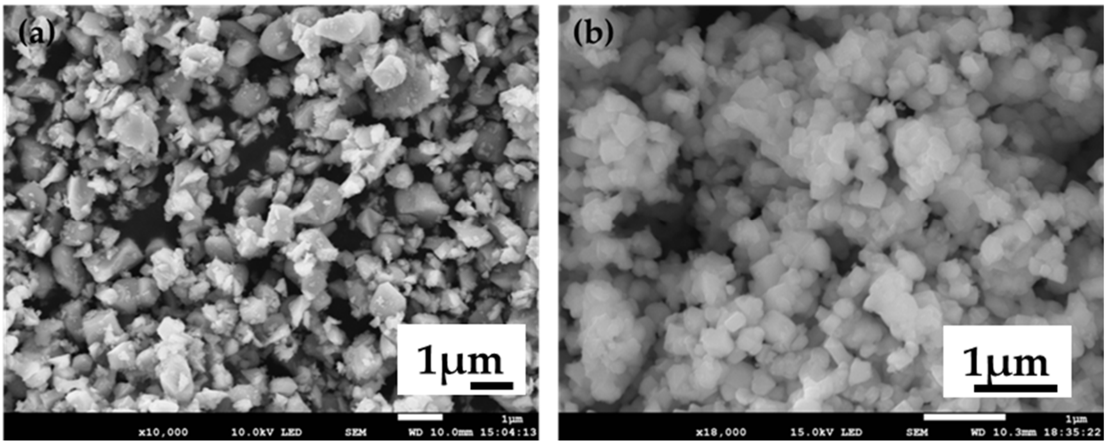

Figure S2. SEM morphology of BZCY(54)$_{8/9}$2 powders (**a**) Purchased powder from KUSAKA RARE METAL PRODUCTS Co., LTD, Japan, as calcined at 1200 °C (**b**) In house synthesized after calcination at 1300 °C and milling.

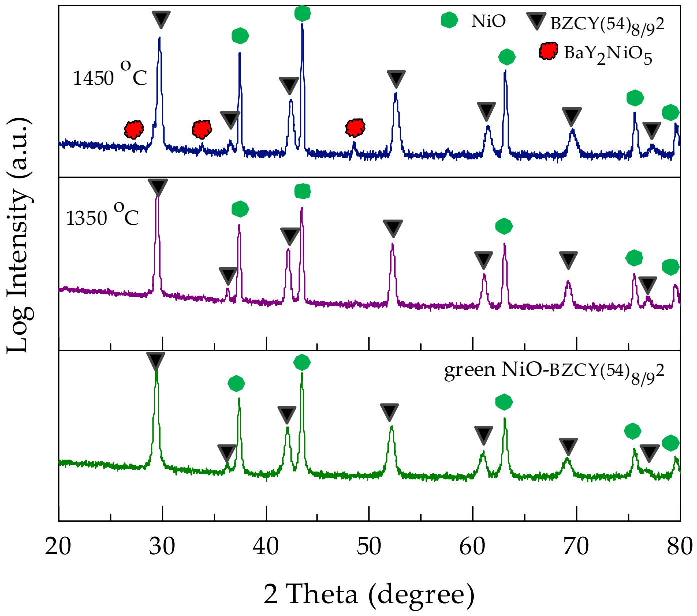

Figure S3. XRD patterns of green NiO-BZCY(54)$_{8/9}$2 electrode substrate and after sintering at 1350 and 1450 °C
